# Spontaneous subdural haematoma in a neonate requiring urgent surgical evacuation

**DOI:** 10.1007/s00701-020-04570-9

**Published:** 2020-09-13

**Authors:** Phillip Correia Copley, Bethan Dean, Angela L. Davidson, Michael Jackson, Drahoslav Sokol

**Affiliations:** 1 Department of Neurosurgery, Royal Infirmary of Edinburgh, 50 Little France Crescent, Edinburgh, EH16 4TJ UK; 2 Department of Neonatology, Royal Infirmary of Edinburgh, 51 Little France Crescent, Edinburgh, EH16 4SA UK; 3 Department of Pediatric Radiology, Royal Hospital for Sick Children, 9 Sciennes Rd, Edinburgh, EH9 1LF UK

**Keywords:** Caesarean section, Craniotomy, Neonate, Spontaneous, Subdural haematoma

## Abstract

We describe the unusual case of a clinically significant subdural haematoma without any underlying cause in a term baby delivered by an elective caesarean section, which required surgical evacuation. We review the literature and describe the presentation, investigation and management options in infants with this infrequent condition.

## Introduction

The incidence of clinically apparent subdural haematomas in infants is estimated to be 24.1/100,000 [5]. Whilst subdural haematomas may occur due to peripartum trauma, occurrence after elective caesarean section (CS) and requirement for surgical intervention is extremely rare.

## Case report

### History and examination

A female infant was born at 39 + 3 weeks by elective CS due to previous forceps delivery. Anomaly scan at 20 weeks was normal. There were no complications during pregnancy. Her birth weight was 2860 g and occipital frontal circumference 33 cm (both 9th–25th centiles). She required no resuscitation, had APGAR score of 9^1^ + 9^5^ and normal cord gases. Intramuscular vitamin K was given. She was reviewed at 18 h due to jaundice. Observations were normal, and examination demonstrated normal tone/reflexes and a soft fontanelle. Her total bilirubin was 80 umol/L (below threshold for phototherapy), haemoglobin 178 g/L and platelets 181 × 10^9^/L.

**Fig. 1:**
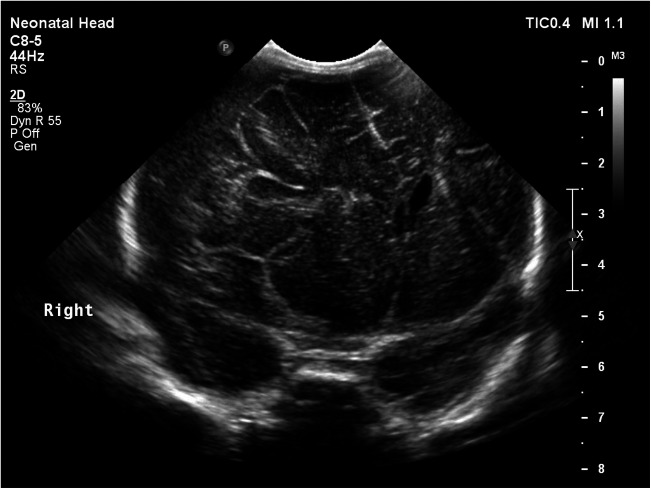
Coronal ultrasound image showing increased echogenicity of large right-sided subdural haematoma causing marked mass effect with midline shift to the left and effacement of the right lateral ventricle

Upon routine newborn examination (30-hours post-delivery), the right pupil was noted to be dilated and non-reactive. The left was normal. The anterior fontanelle was full. There was no abnormality of facial/limb movements, and she was responsive to handling. Ultrasound demonstrated a right subdural haemorrhage (Fig. 1). Repeat bloods showed haemoglobin 182 g/L, platelets 182 × 10^9^/L and a normal coagulation screen. Urgent transfer to the local neurosurgical centre was arranged.

**Fig. 2:**
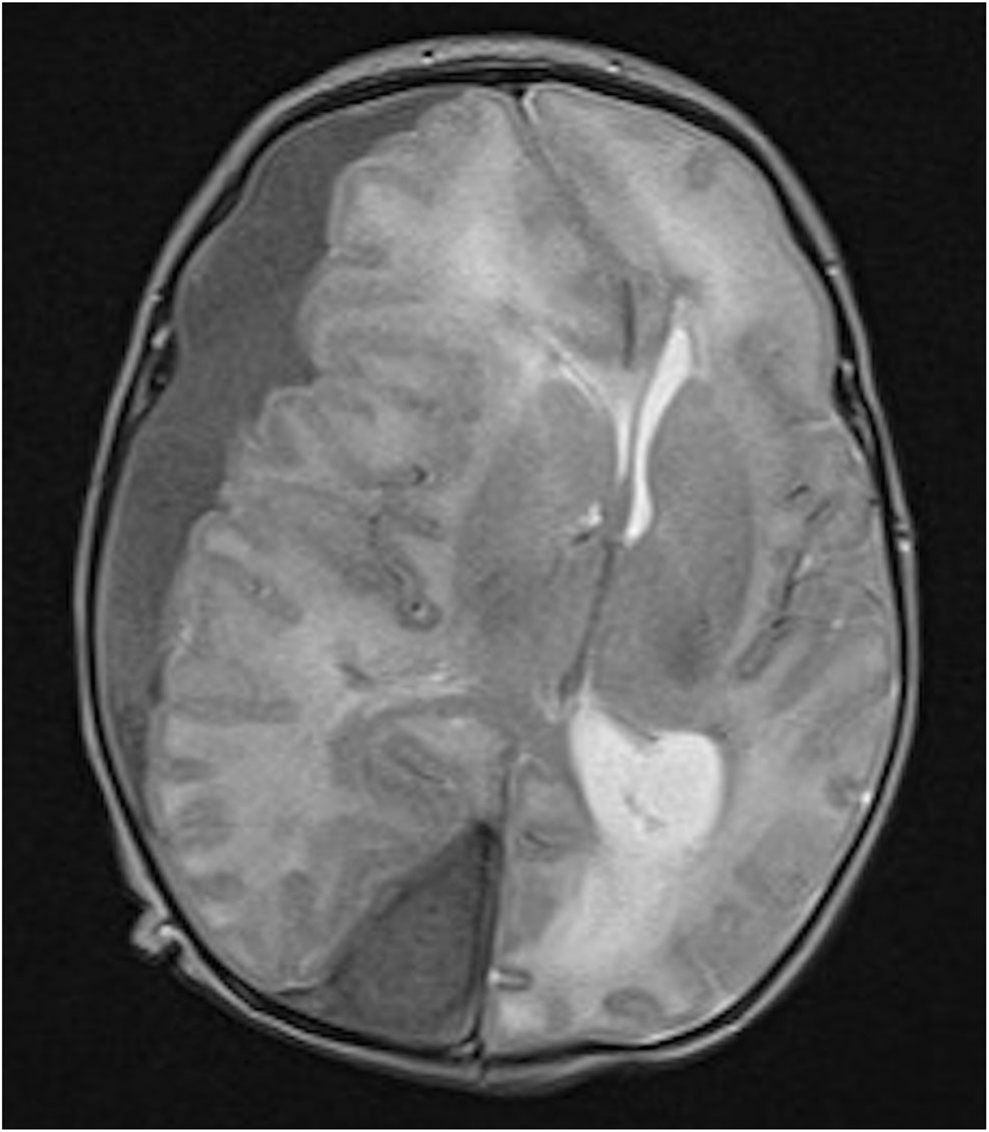
T2-weighted axial MRI. Large right-sided subdural haematoma with severe midline shift. In addition to effacement of the right lateral ventricle, there is enlargement of the trigone of the left lateral ventricle consistent with left-sided hydrocephalus

**Fig. 3:**
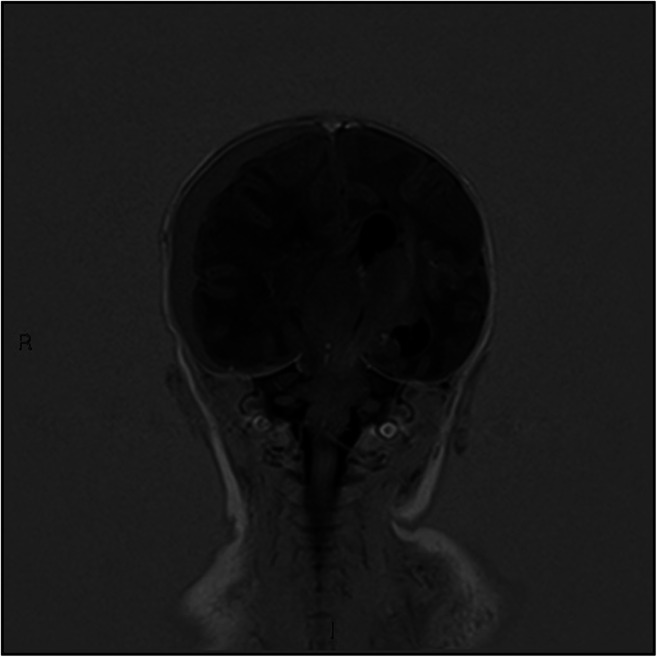
T1-weighted inversion recovery coronal MRI. Large right-sided subdural haematoma with mass effect, midline shift, left-sided hydrocephalus and right-sided uncal herniation

On admission, the only abnormal clinical findings were an isolated right third nerve palsy and a full fontanelle. Under usual circumstances, computed tomography (CT) scan would be performed; however, on this occasion, we had a magnetic resonance imaging (MRI) available immediately. After multidisciplinary discussions, taking into consideration that the baby overall was very well, feeding and stable, we decided on elective intubation and MRI including angiography/venography to assess for possible underlying structural abnormalities (Figs. 2 and 3). No cause was found; however, given the characteristics of the haematoma, a source of bleeding was deemed likely in the occipital inter-hemispheric area. Fontanelle tap of the ventricle to control intracranial pressure (ICP) would likely promote more subdural bleeding. Given the acute nature of the bleed, the haematoma was presumed to be thick/clotted, and we suspected that percutaneous drainage would be ineffective. In theory, conservative management, with further observation, could have been chosen, despite the third nerve palsy, as the baby was otherwise doing well, but risked further bleeding and mass effect, which may have proven fatal. To better control the bleed, mini-craniotomy seemed the best option given the acute nature of the bleed on imaging. The different management strategies were discussed with the parents, and a decision was made to proceed with surgery.

### Operation

Under general anaesthesia, in the supine position, a curvilinear incision was made along the ipsilateral coronal suture. Wide draping allowed extension to the occipital area if needed. Periosteum was left intact over the bone and suture. The bone was removed to form a semi-circular craniotomy with its base on the suture. The bone in neonates is very thin and can be cut with scissors and removed using a rongeur/artery clip, or the craniotome can be used for the sake of speed. The bone was pivoted forward, leaving its attachment to the suture intact. The dura was tense, and after opening, surprisingly, dark liquid blood was encountered under pressure resembling subacute/chronic subdural haematoma with some solid clots and then coming later during washout. Further solid clot was encountered occipitally and left in situ as it was the presumed origin of the bleeding. The dura was closed in a watertight fashion and tacked-up to the bone, which was placed back in its native position. The remaining occipital inter-hemispheric haematoma and sealant agent mass were found unchanged on immediate post-operative imaging, as expected.

### Post-operative course

The baby made an excellent recovery. There was no discernible neurological deficit, and her right pupil was reacting to light. She was discharged home after 6 days. A repeat MRI (including angiography/venography) was performed 2 months later and showed complete resolution of the subdural haematoma with no parenchymal abnormality, normal vasculature and no underlying cause. Nine months post-operatively, she continues to demonstrate normal development.

## Discussion

Subdural haematomas occurring in the peripartum period are almost always caused by the trauma of birth and are usually asymptomatic. Indeed, MRI immediately post-partum demonstrates that 8–46% of infants have asymptomatic subdural collections [12, 20, 25]. Symptomatic subdural haematomas are much more rare, occurring in 16.5/100,000 infants under the age of 1 year [6]. Whilst such cases have been described following emergency/complicated CS, incidence following uncomplicated elective CS is extremely rare [2, 12]. The available evidence is summarized in Table 1.

**Table 1:** Summary of published cases of SDH after a caesarean-section (CS) in term neonates (> 37 weeks of gestation as defined by the American College of Obstetricians and Gynaecologists (https://www.acog.org/clinical/clinical-guidance/committee-opinion/articles/2013/11/definition-of-term-pregnancy))

Article	Study description	Location of SDH	Clinical significance	Cause of SDH	Treatment for SDH	Outcome
MacDonald et al. [14]	Case report after elective CS	Bilateral supratentorial	Symptomatic	Unclear—noted to be severely anaemic	Serial bilateral percutaneous subdural taps	Only mild hypotonia noted at 6 months with normal head circumference
Menezes et al. [16]	Case report after emergency CS due to arrested 2nd stage of labour	Posterior fossa	Symptomatic	Suspected trauma of intrauterine moulding of cranium during prolonged labour prior to CS	Craniotomy and ventriculoperitoneal shunt (VPS)	Normal neurological examination at 16 months of age
Morgan et al. [17]	Case report after emergency CS due to foetal distress	Unilateral supratentorial	Symptomatic	Thrombocytopenia	Percutaneous subdural taps	At 2 years had developmental quotient of 50 and left hemiparesis
Gunn et al. [4]	1 case report of CS due to cephalo-pelvic disproportion and 1 case report of emergency CS for foetal distress	Both bilateral supratentorial	Symptomatic	1. Unclear. Possible trauma of cephalo-pelvic disproportion. Prenatal hydrocephalus noted 2. Unclear Both born to Pacific islander parents and possibility of intrauterine trauma raised by authors as there is practice of abdominal massage during pregnancy in this culture	1. Conservative 2. Evacuated—unclear how	1. Hypotonic quadraparesis, optic nerve atrophy and developmental delay 2. Microcephaly, severe developmental delay and spastic quadraparesis at 3 years old
Franklin et al. [3]	Case report of emergency CS for mild foetal bradycardia	Posterior fossa	Symptomatic	Unknown, although the mother sustained mild abdominal trauma 4 weeks prior to birth	Craniotomy and VPS	Reported to be normal at 3 months
Atluru et al. [1]	Case report of emergency CS after prolonged labour	Bilateral supratentorial	Symptomatic	Coagulopathy	Serial bilateral percutaneous subdural taps. At 10 weeks, bilateral craniotomies and membrane stripping performed, complicated by post-op tension pneumocephalus requiring percutaneous needle aspiration and then bilateral subduro-peritoneal shunts	Seizures and severely abnormal neurological development
Whitby et al. [25]	MRI performed within 48 h following 16 elective and 11 emergency CS in asymptomatic neonates	N/A	None had SDH	N/A	N/A	N/A
Usul et al. [23]	Case report of emergency CS following oblique presentation	Posterior fossa	Symptomatic	None found	Conservative	Radiological resolution on follow-up imaging at 5 months
Looney et al. [12]	MRI performed after 1–5 weeks of life following 23 CS in asymptomatic neonates	N/A	None had SDH	N/A	N/A	N/A
Powers et al. [18]	Case report of an uncomplicated CS	Bilateral supratentorial	Symptomatic	VATER syndrome and macrocephaly	- Serial unilateral percutaneous subdural taps - VPS for hydrocephalus - Represented with shunt infection and reaccumulation of subdural collection noted. Treated with external drainage and antibiotics - Required a subdural catheter to be connected to a new VPS 2 weeks later	Normal neurological examination reported at 1 month of age (prior to insertion of first VPS)
Rooks et al. [20]	MRI performed within 72 h of birth following 13 elective and 19 emergency CS in asymptomatic neonates	All supratentorial	4 had SDH (1 after elective and 3 after emergency CS) All asymptomatic	Elective case had macrosomia All emergency cases had prolonged labour and one required vacuum assistance	Conservative	All resolved by 4 weeks
Tavil et al. [22]	Case series of 16 cases of peripartum intracranial haemorrhage in term newborns	Unilateral supratentorial	1 had SDH after CS Symptomatic	Coagulopathy	VPS	Major physical and cognitive disability
Ma et al. [13]	Case report of elective CS performed due to maternal coagulopathy	Unilateral supratentorial with IVH	Symptomatic	Maternal ingestion of bromadiolone	Conservative	Died
Högberg et al. [6]	Population-based registry study of subdural haematoma in neonates 0–6 days of age born in Sweden from 1997-2014	Not specified	Reported 2 cases after planned CS and 8 cases after emergency CS All symptomatic	Not specified	Not specified	Not specified

Presenting symptoms are due to focal neurological deficit or raised ICP. The skull’s elasticity allows some tolerability to increased pressure; therefore, clinical signs may be subtle, such as poor feeding, irritability and frequent vomiting. On examination, scalp veins may be distended and the anterior fontanelle full. Focal neurological deficits may manifest with weakness or cranial nerve deficits. Papilloedema does not usually develop in neonates, but retinal venous engorgement may be noted [19]. If compression of the brainstem occurs, bradycardia and apnoeic spells may manifest.

Ultrasound provides a rapid non-invasive method for diagnosis and can be done at the bedside. CT is also rapid and usually readily available. However, a significant dose of radiation poses long-term implications. MRI does not deliver radiation and provides exquisite images of the brain but can be time-consuming and not as readily available.

It is imperative to assess for potential underlying causes, such as trauma, coagulopathy, meningitis, cerebral venous thrombosis, metabolic diseases or structural lesions (including tumours or vascular malformations) [7–11, 21]. If time permits, MRI is the most helpful imaging tool in ruling out structural causes. Additional sequences that afford an assessment of the cerebral vasculature are vital. This informs best management and likely prognosis, guiding shared decision-making with the patient’s parents/guardians. In this case, a multidisciplinary decision was made to pursue MRI rather than CT in view of these benefits (without significant time penalty), but we emphasize that in most situations, CT remains the imaging of choice for acute intracranial haemorrhage due to its rapidity.

Management of these cases is based upon the patient’s clinical state, the size of the collection and the preference of the surgeon/parents/guardians [15]. A conservative approach could have been considered given her clinical state, but this could have led to sudden rising ICP and deterioration. Subdural transcutaneous puncture or external subdural drainage allows relief of pressure, if the viscosity of the collection permits; however, there is a risk of infection. Neither of these procedures tends to achieve haematoma resolution but can be useful temporizing methods. In a study of 184 children with subdural haematomata in children under 2 years old, Melo et al. [15] found that despite these procedures, 82% and 50% of children, respectively, required further surgery due to recurrent/residual collection. Shunts provide an option to divert the haematoma but again may be ineffective if the clot is thick. Shunting to the subgaleal space may be employed in children under 3 months old as it negates opening the abdomen. Shunting to the peritoneum may be employed in older infants, typically with a valveless system to encourage drainage. The complication rate for shunting in such cases is around 25%, including the risk of recurrent subdural haematoma upon removal, due to tethering of the proximal catheter [15]. Craniotomy achieves immediate access to the subdural space and evacuation of the clot in order to relive associated mass effect. It also allows evaluation of the brain surface to assess for and control any bleeding source. Given the small circulating blood volume in neonates, the risk of blood loss and death are significant.

If an underlying cause for the haemorrhage is demonstrated on imaging, immediate surgical intervention could lead to catastrophic bleeding and prove fatal. The underlying cause may require treatment prior to surgical management, for example, correction of an underlying coagulopathy or aneurysm [7]. Once we had ruled out any underlying cause and decided to perform a craniotomy, the plan was to perform as small a craniotomy as possible and remove the convexity haematoma. We aimed to leave the presumed clot in the posterior inter-hemispheric fissure alone in order not to provoke further bleeding but draped and planned to extend the unicoronal incision posteriorly along the midline in case of uncontrollable bleeding. Fortunately, this was not required; nevertheless, having bailout strategies is vital when operating on neonates.

Long-term outcomes for infants following non-traumatic subdural haematoma are difficult to predict due to a paucity of reported cases with robust follow-up. In cases of non-accidental injury with subdural haematoma, the reported rate of neurological disability is approximately two-thirds [8]. This may be due to the diffuse nature of the brain injury. For subdural haematomas related to accidental trauma, good outcomes are observed in 45–56% of patients [9–11]. Vinchon et al. reported sixteen cases of symptomatic spontaneous/atraumatic subdural haematomata, noting that twelve of these had idiopathic macrocrania and seven arachnoidomegaly [24]. They suggest idiopathic macrocephaly/arachnoidomegaly, and dehydration may underlie some cases of spontaneous subdural haematomata. Although not the case in our patient, this study provides a useful comparison in terms of outcome. Over a mean follow-up of 21.2 months, they reported a Glasgow outcome score (GOS) of 1 (normal life) in 87.5%. Outcomes were significantly better compared with a matched cohort of infants sustaining traumatic injuries [24].

In conclusion, clinically apparent subdural haematoma is an unusual condition in the neonatal period. The vast majority of these are due to birth-related trauma, or non-accidental injury, which must be readily suspected and its possibility evaluated on a case-by-case basis. However, spontaneous subdural haematomas exist, and a thorough search for an underlying cause is imperative. These cases are difficult due to uncertainty regarding optimal management strategy. Moreover, there is limited evidence to guide prognosis, and so in these rare cases, close collaboration between the multidisciplinary teams involved, and the patient’s caregivers, is mandatory to guide best treatment for the individual child.

## Abbreviations

CS: Caesarean section
CSF: Cerebrospinal fluid
CT: Computed tomography
ICP: Intracranial pressure
GOS: Glasgow outcome score
MRI: Magnetic resonance imaging
VPS: Ventriculoperitoneal shunt

## References

[1] Atluru VL, Kumar IR (1987). Intrauterine chronic subdural haematoma with postoperative tension pneumocephalus. Pediatr Neurol.

[2] Audu L, Mukhtar-yola M, Otunete A, Mairami A, Nwatah V, Mahmud M, Ugwuanyi C, Umar A (2015). Unexplained massive subdural haematoma in a newborn delivered by elective caesarian section: a case report. Niger J Paediatr.

[3] Franklin J, Belkin R, Howieson J, Gallo A (1986). Posterior fossa chronic subdural haematoma in the neonate. AJNR Am J Neuroradiol.

[4] Gunn TR, Mok PM, Becroft DM (1985). Subdural haemorrhage in utero. Pediatrics.

[5] Hobbs C, Childs AM, Wynne J, Livingston J, Seal A (2005). Subdural haematoma and effusion in infancy: an epidemiological study. Arch Dis Child.

[6] Hogberg U, Andersson J, Squier W, Hogberg G, Fellman V, Thiblin I, Wester K (2018). Epidemiology of subdural haemorrhage during infancy: a population-based register study. PLoS One.

[7] Iza-Vallejo B, Mateo-Sierra O, Fortea-Gil F, Ruiz-Juretschke F, Martin YR (2009). Acute subdural haematoma secondary to distal middle cerebral artery aneurysm rupture in a newborn infant. J Neurosurg Pediatr.

[8] Jayawant S, Parr J (2007). Outcome following subdural haemorrhages in infancy. Arch Dis Child.

[9] Karandikar S, Coles L, Jayawant S, Kemp AM (2004). The neurodevelopmental outcome in infants who have sustained a subdural haemorrhage from non-accidental head injury. Child Abuse Rev.

[10] Keenan HT, Runyan DK, Marshall SW, Nocera MA, Merten DF (2004). A population-based comparison of clinical and outcome characteristics of young children with serious inflicted and noninflicted traumatic brain injury. Pediatrics.

[11] Keenan HT, Runyan DK, Nocera M (2006). Longitudinal follow-up of families and young children with traumatic brain injury. Pediatrics.

[12] Looney CB, Smith JK, Merck LH, Wolfe HM, Chescheir NC, Hamer RM, Gilmore JH (2007). Intracranial haemorrhage in asymptomatic neonates: prevalence on MR images and relationship to obstetric and neonatal risk factors. Radiology.

[13] Ma M, Zhang M, Tang X, Li Z (2017). Massive neonatal intracranial haemorrhage caused by bromadiolone: a case report. Medicine (Baltimore).

[14] MacDonald JT, Weitz R, Sher PK (1977). Intrauterine chronic subdural haematoma. Arch Neurol.

[15] Melo JR, Di Rocco F, Bourgeois M, Puget S, Blauwblomme T, Sainte-Rose C, Meyer PG, Zerah M (2014). Surgical options for treatment of traumatic subdural haematomas in children younger than 2 years of age. J Neurosurg Pediatr.

[16] Menezes AH, Smith DE, Bell WE (1983). Posterior fossa haemorrhage in the term neonate. Neurosurgery.

[17] Morgan ME, Hensey OJ, Cooke RW (1983). Convexity cerebral haemorrhage in the neonate: in vivo ultrasound diagnosis. Arch Dis Child.

[18] Powers CJ, Fuchs HE, George TM (2007). Chronic subdural haematoma of the neonate: report of two cases and literature review. Pediatr Neurosurg.

[19] Rigi M, Almarzouqi SJ, Morgan ML, Lee AG (2015). Papilledema: epidemiology, etiology, and clinical management. Eye Brain.

[20] Rooks VJ, Eaton JP, Ruess L, Petermann GW, Keck-Wherley J, Pedersen RC (2008). Prevalence and evolution of intracranial haemorrhage in asymptomatic term infants. AJNR Am J Neuroradiol.

[21] Silva AHD, Gander L, Wijesinghe H, Rodrigues D (2019) Spontaneous neonatal subdural haemorrhage: always non-accidental injury? Br J Neurosurg:1–4. 10.1080/02688697.2019.1694134 10.1080/02688697.2019.1694134 31771378

[22] Tavil B, Korkmaz A, Bayhan T, Aytac S, Unal S, Kuskonmaz B, Yigit S, Cetin M, Yurdakok M, Gumruk F (2016). Foetal and neonatal intracranial haemorrhage in term newborn infants: Hacettepe University experience. Blood Coagul Fibrinolysis.

[23] Usul H, Karaarslan G, Cakir E, Kuzeyl K, Mungan L, Baykal S (2005). Conservative management of spontaneous posterior fossa subdural haematoma in a neonate. J Clin Neurosci.

[24] Vinchon M, Delestret I, DeFoort-Dhellemmes S, Desurmont M, Noule N (2010). Subdural haematoma in infants: can it occur spontaneously? Data from a prospective series and critical review of the literature. Childs Nerv Syst.

[25] Whitby EH, Griffiths PD, Rutter S, Smith MF, Sprigg A, Ohadike P, Davies NP, Rigby AS, Paley MN (2004). Frequency and natural history of subdural haemorrhages in babies and relation to obstetric factors. Lancet.

